# Inhibition of SRPK1, a key splicing regulator, exhibits antitumor and chemotherapeutic-sensitizing effects on extranodal NK/T-cell lymphoma cells

**DOI:** 10.1186/s12885-022-10158-6

**Published:** 2022-10-27

**Authors:** Cuiying He, Beichen Liu, Huan-You Wang, Lili Wu, Guimin Zhao, Chen Huang, Yueping Liu, Baoen Shan, Lihong Liu

**Affiliations:** 1 Department of Hematology, The Fourth Hospital of Hebei Medical University, NO.169, TianShan Street, Shijiazhuang, 050035 Hebei China; 2 Hebei Provincial Key Laboratory of Tumor Microenvironment and Drug Resistance, Shijiazhuang, China; 3 Hebei Medical University, Shijiazhuang, China; 4 Department of Pathology, University of California San Diego, San Diego, CA USA; 5 Department of Pathology, The Fourth Hospital of Hebei Medical University, Shijiazhuang, China; 6 Research Center and Tumor Research Institute, The Fourth Hospital of Hebei Medical University, Shijiazhuang, China

**Keywords:** ENKTL, SRPK1, Cisplatin resistance, Apoptosis, ATF4/CHOP/AKT1

## Abstract

**Background:**

Increasing evidence has convincingly shown that abnormal pre-mRNA splicing is implicated in the development of most human malignancies. Serine/arginine-rich protein kinase 1 (SRPK1), a key splicing regulator, is reported to be overexpressed in leukemia and other cancer types, which suggests the therapeutic potential of targeting SRPK1.

**Methods:**

SRPK1 expression was measured in 41 ENKTL patients by immunohistochemistry and mRNA expression was analyzed by qRT‒PCR. We knocked down SRPK1 expression in the ENKTL cell line YT by siRNA transfection and inhibited SRPK1 using inhibitors (SPHINX31 and SRPIN340) in YT cells and peripheral blood lymphocytes (PBLs) isolated from ENKTL patients to investigate its role in cell proliferation and apoptosis. Then, RNA-seq analysis was performed to predict the potential signaling pathway by which SRPK1 inhibition induces cell death and further verified this prediction by Western blotting.

**Results:**

In the present study, we initially evaluated the clinical significance of SRPK1 in extranodal natural killer/T-cell lymphoma (ENKTL), a very aggressive subtype of non-Hodgkin lymphoma. The expression of SRPK1 in ENKLT patients was examined by immunohistochemistry and qRT‒PCR, which revealed SRPK1 overexpression in more than 60% of ENKTL specimens and its association with worse survival. Cellular experiments using the human ENKTL cell line YT and PBLs from ENKTL patients, demonstrated that inhibition of SRPK1 suppressed cell proliferation and induced apoptosis. Subsequently, we investigated the downstream targets of SRPK1 by RNA-seq analysis and found that SRPK1 inhibition induced ATF4/CHOP pathway activation and AKT1 inhibition. Furthermore, ENKTL patients presenting high SRPK1 expression showed resistance to cisplatin-based chemotherapy. The association of SRPK1 expression with cisplatin resistance was also confirmed in YT cells. SRPK1 overexpression via pLVX-SRPK1 plasmid transfection dramatically decreased the sensitivity of YT cells to cisplatin, while siRNA-mediated SRPK1 knockdown or SRPK1 inhibitor treatment significantly increased cisplatin cytotoxicity.

**Conclusion:**

In summary, these results support that SRPK1 might be a useful clinical prognostic indicator and therapeutic target for ENKTL, especially for patients who relapse after cisplatin-based chemotherapies.

**Supplementary Information:**

The online version contains supplementary material available at 10.1186/s12885-022-10158-6.

## Introduction

Extranodal natural killer/T-cell lymphoma (ENKTL) is an aggressive type of peripheral T-cell lymphoma (PTCL) originating from normal natural killer (NK) cells or cytotoxic T cells. Although chemotherapy and radiotherapy improve the clinical outcomes of patients with early-stage disease, the overall survival (OS) of patients with advanced ENKTL remains poor because of frequent relapse or resistance to treatment [1, 2]. Currently, no highly effective targeted therapies are available for this aggressive lymphoma.

The majority of human genes contain multiple protein-coding regions, known as exons, that are separated by noncoding intervening sequences known as introns. RNA alternative splicing refers to the process of excising noncoding introns and splicing exons to produce mature mRNA, which gives rise to numerous proteins from a relatively limited numbers of genes. A growing number of studies have convincingly confirmed that aberrant RNA splicing is associated with cancer initiation [3–5]. RNA splicing occurs in the spliceosome and is tightly regulated by a variety of splicing factors, including the serine/arginine-rich (SR) protein family [6, 7]. SR proteins are extensively phosphorylated at their RS domains by multiple kinases, and this process is mediated by the predominant enzymes SR-specific protein kinases (SPRKs) [8, 9]. SRPKs, which commonly include SRPK1 and SRPK2, are typically localized in the cytoplasm and translocated to the nucleus when activated by upstream signals to regulate splicing [10]. SRPK1, the first cloned and characterized SRPK, has been reported to be a major regulator of alternative splicing. The critical role of SRPK1 in malignancies has been reported in several human cancers, including lung, breast, prostate, cervical, colorectal, stomach, and liver cancers [11–16].

Recent studies have revealed that SRPK1 promotes oncogenesis through overexpression and functional alterations, which indicates the therapeutic potential of targeting this protein [17]. For example, overexpression of the SRPK1 protein in cancer cells and its autologous antibody in plasma was observed in patients with acute-type of adult T-cell leukemia [18]. Tzelepis *et al.* demonstrated that SRPK1 inhibition affects the transcript levels of many genes, and that SRPK1 may be a plausible therapeutic target in acute myeloid leukemia (AML) [19, 20]. These studies suggest that SRPK1 may be important in hematologic malignancies and its potential use for developing therapeutic strategies. However, no studies have investigated the status of SRPK1 expression in ENKTL patients and the possibility of targeting SRPK1 as an ENKTL therapeutic strategy.

In this study, we reported for the first time that SRPK1 was expressed in more than 60% of ENKTL patients. Furthermore, we found that high expression of SRPK1 was associated with poor outcomes and cisplatin resistance. The association of SRPK1 expression with cisplatin resistance was also confirmed in the ENKTL cell line. In addition, we demonstrated that SRPK1 inhibitors or silencers suppressed the proliferation of ENKTL cells and induced apoptosis, which may potentially be achieved via the activation of the ATF4/CHOP pathway and inhibition of AKT1 expression. These data indicate that SRPK1 is a valuable prognostic biomarker and that its pharmacological inhibition is an alternative therapeutic strategy for ENKTL.

## Methods

### Patient selection

Pathological archives were searched for patients diagnosed with ENKTL between 2015 and 2021 at the Fourth Hospital of Hebei Medical University. Forty-one patients were determined to have archival formalin-fixed paraffin-embedded (FFPE) tissues obtained by diagnostic biopsies performed pretreatment that was available for immunohistochemistry (IHC). The diagnosis of ENKTL was made according to the criterion outlined by the revised 4^th^ edition of the WHO Classification of Tumors of Hematopoietic and Lymphoid Tissue [21].

### IHC for SRPK1

IHC was performed on FFPE tissue sections using a Leica RM2245 - Semi Motorized Rotary Microtome (Leica, Germany). The anti-SRPK1 antibody (dilution 1:100) was purchased from BD Biosciences (BD Biosciences, San Jose, CA, USA). The intensity (I) of IHC staining was defined as follows: 0 = no staining at all; 1 = weak staining; and 2 = strong staining. The percentage (%) of staining was defined as the percentage of positively stained cells as follows: 0 = no positive cells; 1 = 1–49%; and 2 = 50–100%. The total IHC score was calculated by multiplying I and % (I x %) and ranged from 0 to 4. The total IHC score for each case was recorded blindly without any information, including treatment knowledge. A slide was recorded as negative for SRPK1 expression when the total IHC score was ≤ 1 and defined as positive when the total IHC score was ≥ 2.

### SRPK1 mRNA extraction and qRT‒PCR

Thirty-one out of 41 specimens with adequate tissue were used for mRNA extraction. The FFPE RAN Kit (OMEGA Biotek) was used for RNA extraction from the FFPE tissue sections. Then, cDNA was prepared from total RNA (1 μg) using the FastQuant RT Kit (Tiangen, China). qRT‒PCR was performed using the TaqMan® Gene Expression Master Mix (Thermo Fisher Scientific, USA). The Glyceraldehyde-3-phosphate dehydrogenase (GAPDH) was used as the mRNA input control, and relative mRNA expression levels were computed using the 2^-Δ(CT)^ method. The primer sequences are shown below:

- SRPK1-F: 5′-GCAACAGAATGGCAGCGATC-3′,
- SRPK1-R: 5′-CTGGCGCTTCTGCTTCTTC-3′,
- GAPDH-F: 5′-CCTGCACCACCAACTGCTTA-3′,
- GAPDH-R: 5′-ATGGCATGGACTGTGGTCATG-3′.

### Cell culture and chemicals

The human ENKTL cell line YT was kindly provided by Prof. Mingzhi Zhang, Zhengzhou University First Affiliated Hospital, Zhengzhou, China. YT cells were cultured in Roswell Park Memorial Institute 1640 medium (RPMI1640, Gibco, USA) containing 10% fetal bovine serum in a humidified 5% CO_2_ incubator at 37 °C. The SRPK1 inhibitors SPHINX31 and SRPIN340 were purchased from MedChemExpress USA (Monmouth Junction, NJ, USA).

### Peripheral blood lymphocyte (PBL) isolation

PBLs were isolated from ENKTL patients using Human Peripheral Blood Lymphocyte Separation Solution (Tbdscience, China). All participants provided written informed consent, and the procedure was performed in accordance with the Ethics Committee of the Forth Hospital of Hebei Medical University.

### Cell viability assay

Cell viability, expressed as cell proliferation, was measured using a Cell Counting Kit-8 (CCK-8) assay. YT cells or PBLs were seeded in 96-well plates and cultured with or without SRPK1 inhibitors. CCK-8 solution was added after each culture period. After 2 hours of incubation, the optical density (OD) values at 450 nm were measured by a Multiskan Sky Microplate Spectrophotometer (Thermo Fisher Scientific, USA).

### siRNA, plasmids, and transfection

Downregulation and upregulation of SRPK1 and ATF4 was achieved by small interfering RNAs (siRNAs). si-SRPK1s and si-ATF4s were acquired from Sangon Biotech, China. The selected sequences of siRNAs are as follows:

- si-SRPK1-1: 5′-GUGGCAAUGAAAGUAGUUAAATT-3′,
- si-SRPK1-2: 5′-CAGACUCUUGUACACCUAUAATT-3′,
- si-SRPK1-3: 5′- GCAGCUGGCUUCACAGAUUUCTT-3′,
- si-ATF4-1: 5′-CCUAGGUCUCUUAGAUGAUUATT-3′,
- si-ATF4-2: 5′-GUUGGUCAGUCCCUCCAACAATT-3′,
- si-ATF4-3: 5′-CCUCAGUGCAUAAAGGAGGAATT-3′.

The SRPK1 or AKT1 expression plasmid was generated from the full-length SRPK1 or AKT1 cDNA and cloned in-frame into a PLVX-Puro plasmid (pLVX-SRPK1 or pLVX-AKT1). YT cells were transfected with the siRNA using Lipofectamine 3000 (Invitrogen, Carlsbad, CA, USA). SRPK1 or AKT1 expression plasmid was electrotransfected into YT cells, and cells harboring the pLVX-Puro were selected for puromycin resistance.

### Flow cytometry assay

Annexin V (420404, Biolegend, USA) and 7-amino-actinomycin D (7-AAD, 640920, Biolegend, USA) double staining was performed to detect cell apoptosis. Cells were analyzed using the MACSQuant Analyzer 10 (Miltenyi Biotec, Bergisch Gladbach, Germany), and the results were analyzed by FlowJo software.

### Western blotting assay

YT cells were lysed with RIPA lysis buffer to prepare whole-cell extracts. Equal amounts of cell protein extracts (10 μg) were separated by sodium dodecyl sulfate–polyacrylamide gel electrophoresis (SDS‒PAGE), transferred to and immobilized on polyvinylidene fluoride (PVDF) membranes (Pall, Westborough, MA, USA), and then probed with the appropriate primary and secondary antibodies. Immunodetection was performed using a Bio-Rad ChemiDoc XRS+ System (Hercules, CA, USA). The expression of the target protein was normalized to that of GAPDH. Antibodies against SRPK1 (611072, BD Biosciences), poly-ADP-ribose polymerase (PARP, 9542, Cell Signaling Technology), Caspase-3 (9662, Cell Signaling Technology), ATF-4 (activating transcription factor 4, 11815, Cell Signaling Technology), CHOP (C/EBP homologous protein, 2895, Cell Signaling Technology), phospho-AKT1 (ab81283, Abcam), AKT1 (60203-20I, Proteintech Group), and GAPDH (60004-1-Ig, Proteintech Group) were used.

### RNA-seq analysis

Total RNA was extracted from YT cells using TRIzol reagent (Invitrogen, Carlsbad, CA, USA). RNA-seq was performed using the 2×150 bp paired-end sequencing (PE150) on an Illumina NovaSeq™ 6000 platform (LC-Bio Technology Co., Ltd., Hangzhou, China) according to the manufacturer's instructions. The RNA-Seq results were reported as fragments per kilobase of exon model per million mapped reads (FPKM) values. The original data of RNA-seq data have been submitted to the NCBI SRA database (PRJNA774953).

### Analysis of mRNA sequencing data

Cutadapt software was used to remove the reads that contained adaptor contamination. After removing the low-quality bases and undetermined bases, HISAT2 software was used to map reads to the genome. The mapped reads of each sample were assembled using StringTie with default parameters (command line: ~stringtie -p 4 -Ggenome.gtf -o output.gtf -l sample input.bam). Then, all transcriptomes from all samples were merged to reconstruct a comprehensive transcriptome using gffcompare software. After the final transcriptome was generated, StringTie and ballgown were used to estimate the expression levels of all transcripts and perform expression level for mRNAs by calculating FPKM. The differentially expressed mRNAs with fold change > 2 or fold change < 0.5 and p value < 0.05 were selected by the R package edgeR or DESeq2, and then GO enrichment were performed on the differentially expressed mRNAs.

### Statistical analysis

Survival analysis was conducted using the Kaplan–Meier method, and the results were compared using the log-rank test. OS was calculated from the date of initial diagnosis to the date of death. Progression-free survival (PFS) was calculated from the date of initial diagnosis to disease progression or death from any cause. The association between SRPK expression and the clinical parameters was conducted by Fisher's exact test. An independent samples t test was used to evaluate the mRNA expression in the SRPK1-negative and SRPK1-positive groups. A two-sided *P* value <0.05 was considered to indicate a statistically significant difference. Statistical analyses were performed using SPSS 19.0 software (IBM Corp., Armonk, NY, USA).

## Results

### SRPK1 was expressed in more than half of ENKTL patients

**Fig. 1:**
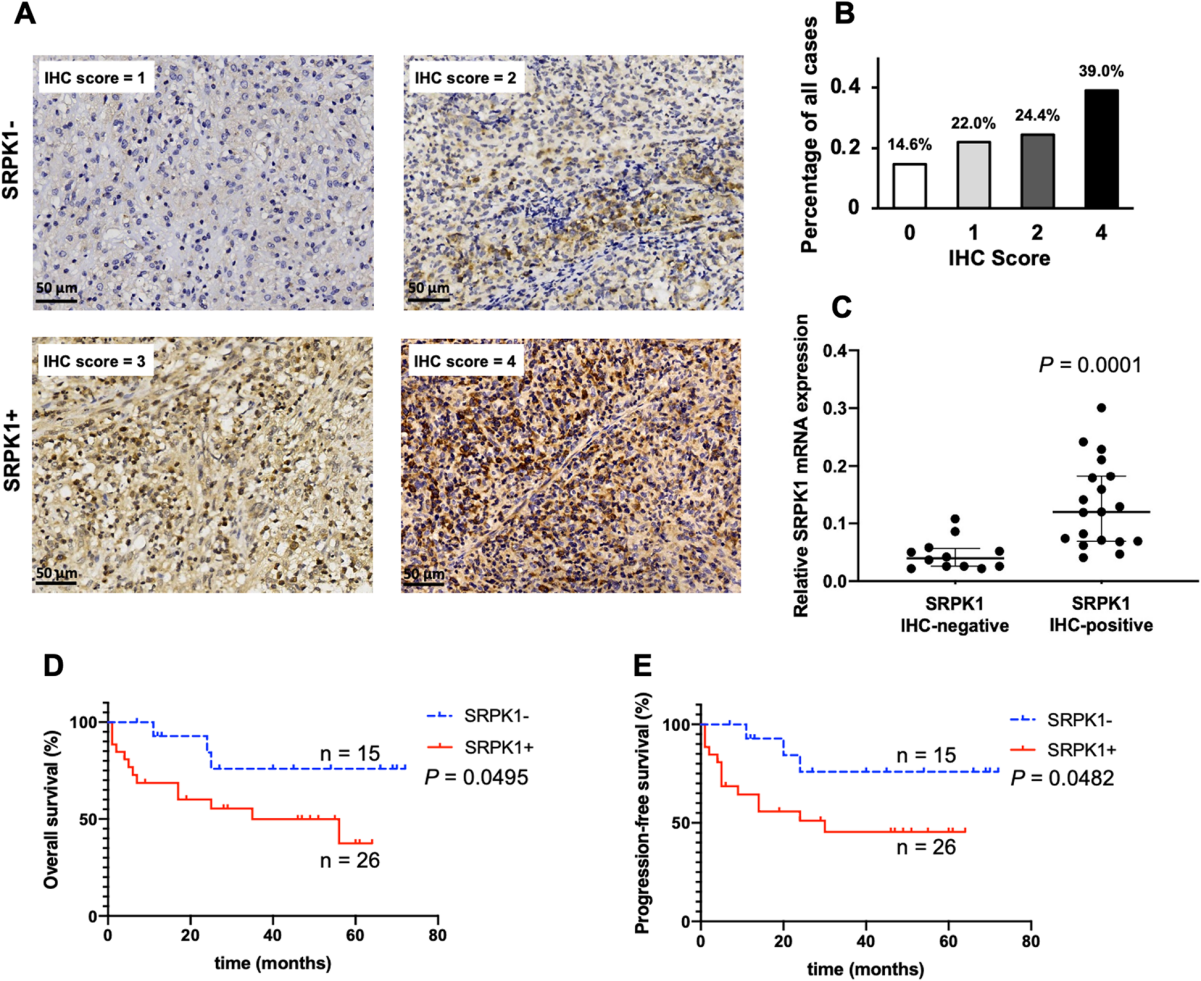
SRPK1 is expressed in more than half of ENKTL patients and is associated with adverse clinical outcomes. **A **Immunohistochemical expression of SRPK1 in NK/T-cell lymphomas. **B** Distribution of SRPK1 expression for different IHC scores. **C** Relative SRPK1 mRNA expression levels in ENKTL patients. **D** and **E** Kaplan‒Meier survival analysis revealed that SRPK1 expression was significantly associated with a shorter OS and PFS

We first examined the expression of SRPK1 in FFPE tissues from 41 ENKTL patients. As shown in Fig. 1A, SRPK1-positive cases showed diffuse or granular cytoplasmic and nuclear staining patterns with variable degrees of intensity. According to the defined IHC scores mentioned above, positive SRPK1 expression was observed in 63.4% (26/41) of the patients (Fig. 1B). To further explore SRPK1 mRNA expression in ENKTL, qRT‒PCR analysis was performed for 31 ENKTL cases. The SRPK1 mRNA levels in 35 SRPK1 IHC-positive tissues were significantly higher than those in 10 SRPK1 IHC-negative samples (Fig. 1C). These results confirmed that SRPK1 was overexpressed in more than half of ENKTL patients.

### Positive expression of SRPK1 was associated with shorter OS and PFS

To explore the potential effects and relationship between the status of SRPK1 expression and clinical outcomes, we performed Kaplan‒Meier analyses of OS and PFS according to SRPK1 status. Patients with positive SRPK1 expression had significantly shorter OS and PFS than those with negative SRPK1 expression (*P*<0.05, Fig. 1D and E). As shown in Table 1, among the laboratory and clinical parameters analyzed, the Ki-67 proliferation index (*P* = 0.018) and serum β2 microglobulin (*P* = 0.017) were significantly higher in patients with positive SRPK1 expression, which may partially explain the poor prognosis. Although elevated serum lactate dehydrogenase (LDH) and bone marrow involvement were more common among SRPK1-positive patients than SRPK1-negative patients, the associations were not statistically significant.

**Table 1:** Comparison of clinical parameters between patients with positive or negative SRPK1 expression

Clinical Parameters	Overall	SRPK1-	SRPK1 +	*P-*value
N	41	15	26	
Gender				0.381
Male	28	12	16	
Female	13	3	10	
Age				0.382
≥ 60	10	2	8	
< 60	31	13	18	
Ann Arbor Stage				0.730
I/II	26	9	17	
III/IV	15	6	9	
Serum LDH (U/l)				0.678
Normal	28	12	16	
Increased	13	3	10	
IPI				0.730
0 ~ 1	26	9	17	
≥ 2	15	6	9	
Ki-67				0.018
< 60	15	9	6	
≥ 60	26	6	20	
Serum β2 microglobulin (μg/ml)				0.017
Normal	20	11	9	
Increased	21	4	17	
B symptoms				0.607
Absent	24	8	16	
Present	17	7	10	
BM involvement				0.727
Yes	8	2	6	
No	33	13	20	
Sensitive to cisplatin				0.033
Yes	11	5	6	
No	13	1	12	

B symptoms: unintentional weight loss of > 10% of normal body weight over a period of six months or less, fever > 38 °C for more than three days without any evident cause other than lymphoma, and human drenching night sweats

Sensitive to cisplatin: Patients achieved complete remission or partial response after cisplatin-containing chemotherapies, which include DDGP regimen chemotherapy (dexamethasone, cisplatin, gemcitabine, and pegaspargase) and IPGDP regimen chemotherapy (ifosfamide, pegaspargase, gemcitabine, cisplatin, and dexamethasone)

### SRPK1 inhibition suppressed cell proliferation

**Fig. 2:**
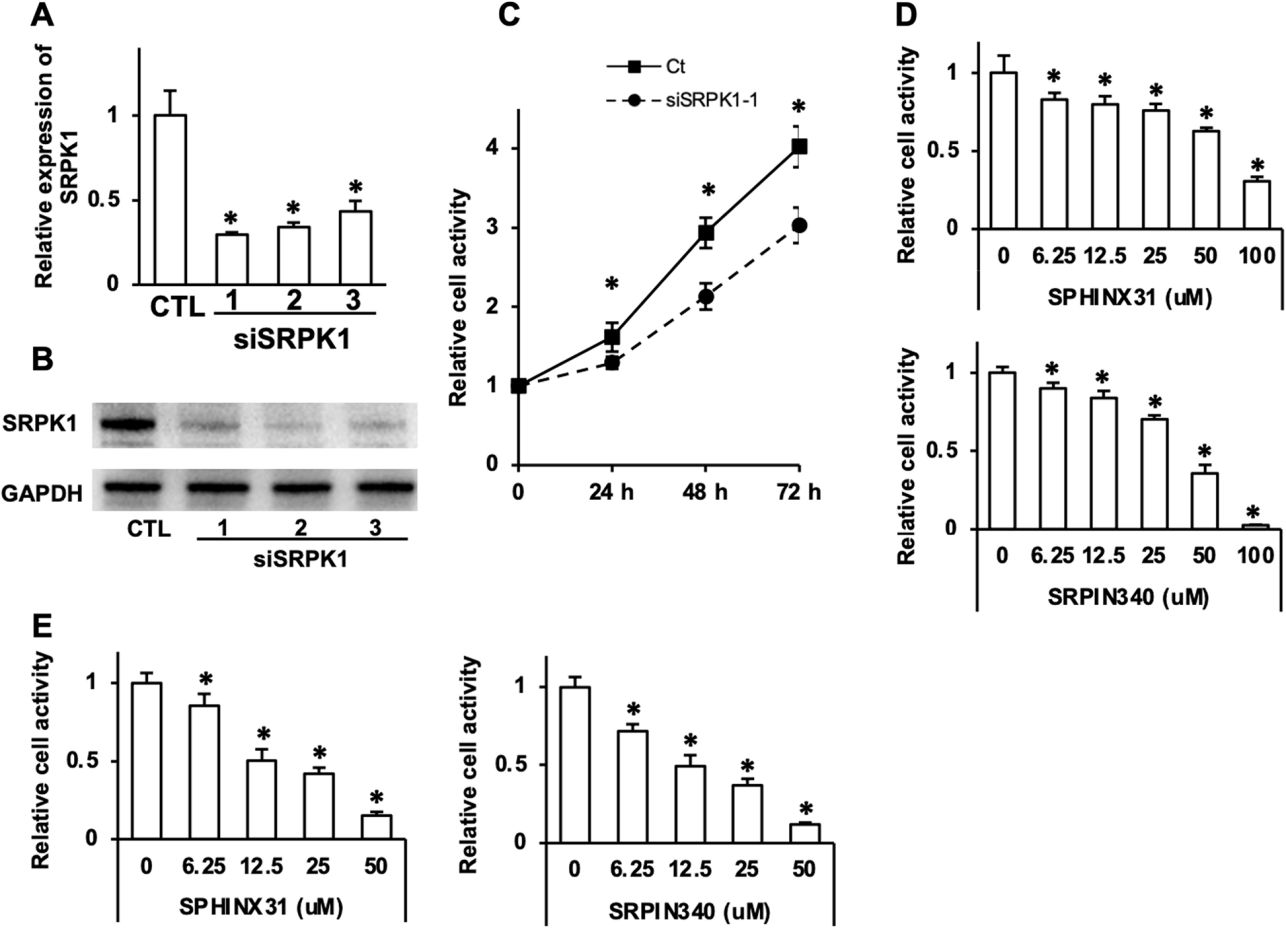
SRPK1 inhibition suppressed the cell proliferation of ENKTL cells. SRPK1 mRNA **(A)** and protein (**B**) levels were downregulated by siSRPK1s in YT cells. **C **siSRPK1-1 decreased YT cell viability for up to 72 hours. **D **Decreased YT cell viability was associated with the SRPK1 inhibitors SPHINX31 and SRPIN340 in a dose-dependent manner. **E **The SRPK1 inhibitors SPHINX31 and SRPIN340 decreased the relative cell activity in PBLs from ENKTL patients in a dose-dependent manner (mean activity ± SD from quadruplicate wells, *P < 0.05 versus the control group)

We next sought to determine whether SRPK1 inhibition could slow the growth of ENKTL cells. In vitro, we successfully downregulated SRPK1 at the mRNA and protein levels in YT cells via si-SRPK1 transfection (Fig. 2A and B). Downregulation of SRPK1 significantly decreased cell viability after up to 72 hours in culture (Fig. 2C). Similarly, the viability of YT cells was significantly reduced when cocultured with SRPK1 inhibitor SPHINX31 or SPRIN340 (Fig. 2D). Furthermore, the proliferation of PBLs from ENKTL patients was also suppressed by SRPK1 inhibitors (Fig. 2E). These results demonstrated that SRPK1 silence and inhibition resulted in the suppression of cell proliferation in ENKTL cells.

### SRPK1 inhibition promoted the apoptosis of ENKTL cells

**Fig. 3:**
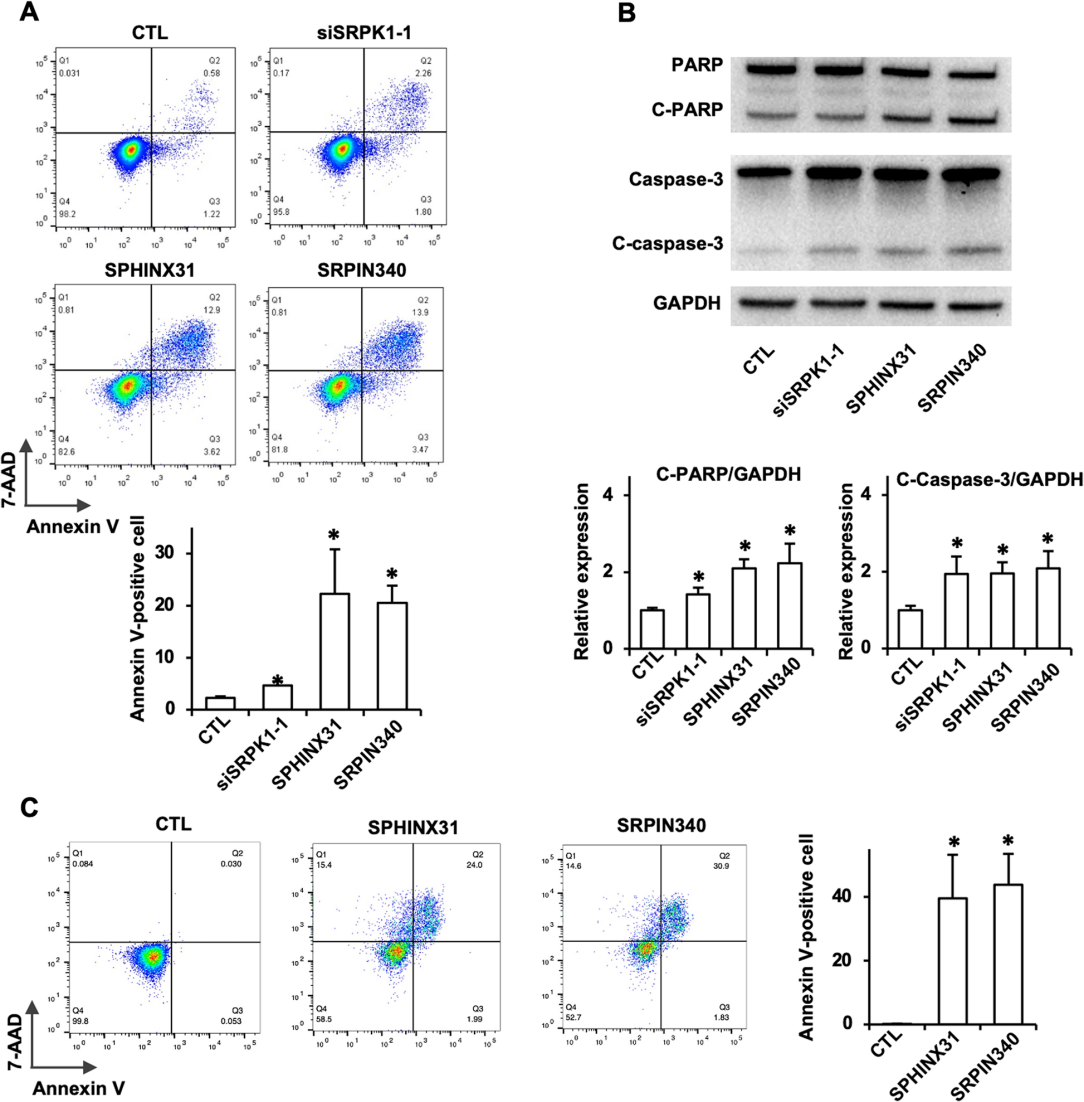
SRPK1 inhibition promoted apoptosis in ENKTL cells. **A** YT cells were transfected with SRPK1 siRNA or treated with SPHINX31 (12.5 μM) or SRPIN340 (12.5 μM) for 24 h. The cells were analyzed by flow cytometry after Annexin V/7-AAD double staining. The percentage of Annexin V-positive cells is presented as the mean ± SD from 3 independent experiments (*P < 0.05). **B** Western blotting of whole-cell extracts from YT cells showed increased levels of cleaved PARP and cleaved caspase-3. The Expression levels of cleaved PARP/GAPDH and cleaved caspase-3/GAPDH are presented as the means ± SDs from 3 independent experiments (*P < 0.05). **C** PBLs from ENKTL patients were treated with SPHINX31 (12.5 μM) or SRPIN340 (12.5 μM) for 24 h, and apoptotic cells were dtected by Annexin V/7-AAD double staining

SRPK1 inhibition-induced apoptotic was then detected by Annexin V/7-AAD double staining in YT cells and PBLs from ENKTL patients. Both SRPK1 inhibitors (SPHINX31 and SRPIN340) and siRNA increased the number of Annexin V-positive cells (Fig. 3A). Moreover, SRPK1 inhibition-induced apoptosis was further confirmed by elevated levels of cleaved PARP and cleaved caspase-3 (Fig. 3B). SRPK1 inhibitor stimulation increased the number of apoptotic cells among PBLs from ENKTL patients (Fig. 3C). The results show that SRPK1 inhibition could suppress cell proliferation and promote apoptosis in ENKTL cells, which indicates that SRPK1 might be a therapeutic target for ENKTL.

### ATF4/CHOP pathway and AKT1 were downstream targets of SRPK1 inhibitors

**Fig. 4:**
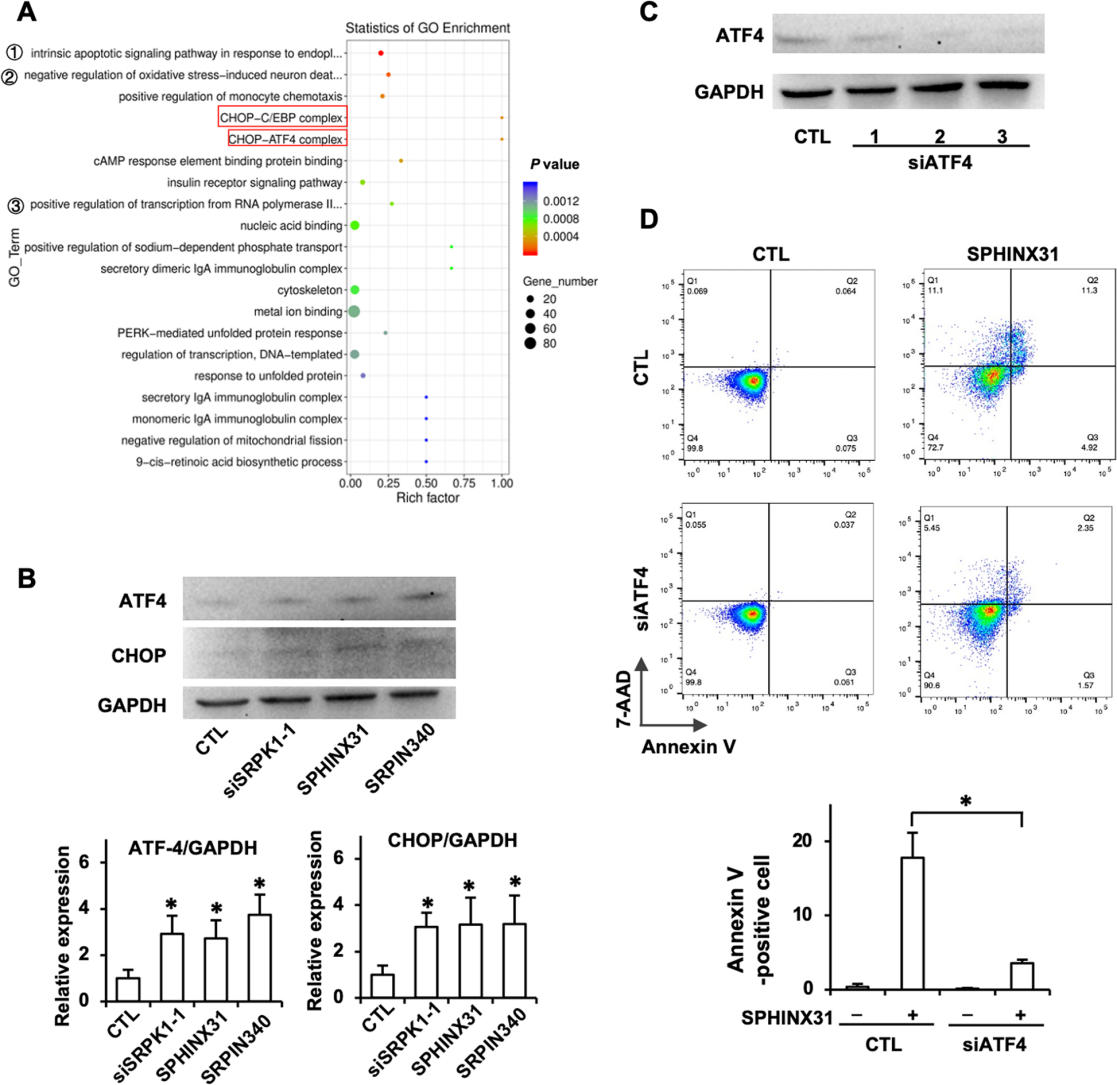
The ATF4/CHOP pathway is a downstream target of SRPK1 inhibition. **A** YT cells were transfected with SRPK1 siRNA or treated with SPHINX31 (12.5 μM) or SRPIN340 (12.5 μM) for 24 h. Gene Ontology (GO) enrichment analysis showed that multiple pathways were affected by SRPK1 downregulation. (①: intrinsic apoptotic signaling pathway in response to endoplasmic reticulum stress; ②: negative regulation of oxidative stress-induced neuron death; ③: positive regulation of transcription from RNA polymerase II promoter in response to endoplasmic reticulum stress). **B** Western blotting showed that CHOP and AFT4 were upregulated by SRPK1 silencing and inhibition. **C** Silencing ATF4 by siRNA transfection decreased the expression of ATF4 in YT cells. **D** Annexin V/7-AAD double staining showed the effect of ATF4 knockdown by siRNA on SPHINX31-induced apoptosis in YT cells.

To investigate downstream target genes most likely affected by SRPK1 inhibition, the differentially expressed genes in SRPK1 knockdown (siSRPK1) and inhibitor (SPHINX31 and SRPIN340)-treated and untreated YT cells were subjected to transcriptome analysis using RNA-seq. First, as shown by Gene Ontology (GO) enrichment analyses, CHOP-C/EBP and CHOP-ATF4 complexes, among others, were enriched (Fig. 4A). The increased expression of CHOP and ATF4 proteins was further confirmed by Western blotting (Fig. 4B). To confirm the involvement of the CHOP-ATF4 pathway in SRPK1 inhibition-induced apoptosis, we suppressed ATF4 expression with si-ATF4 (Fig. 4C). Downregulation of ATF4 expression rescued SPHINX31-induced apoptosis (Fig. 4D).

**Fig. 5:**
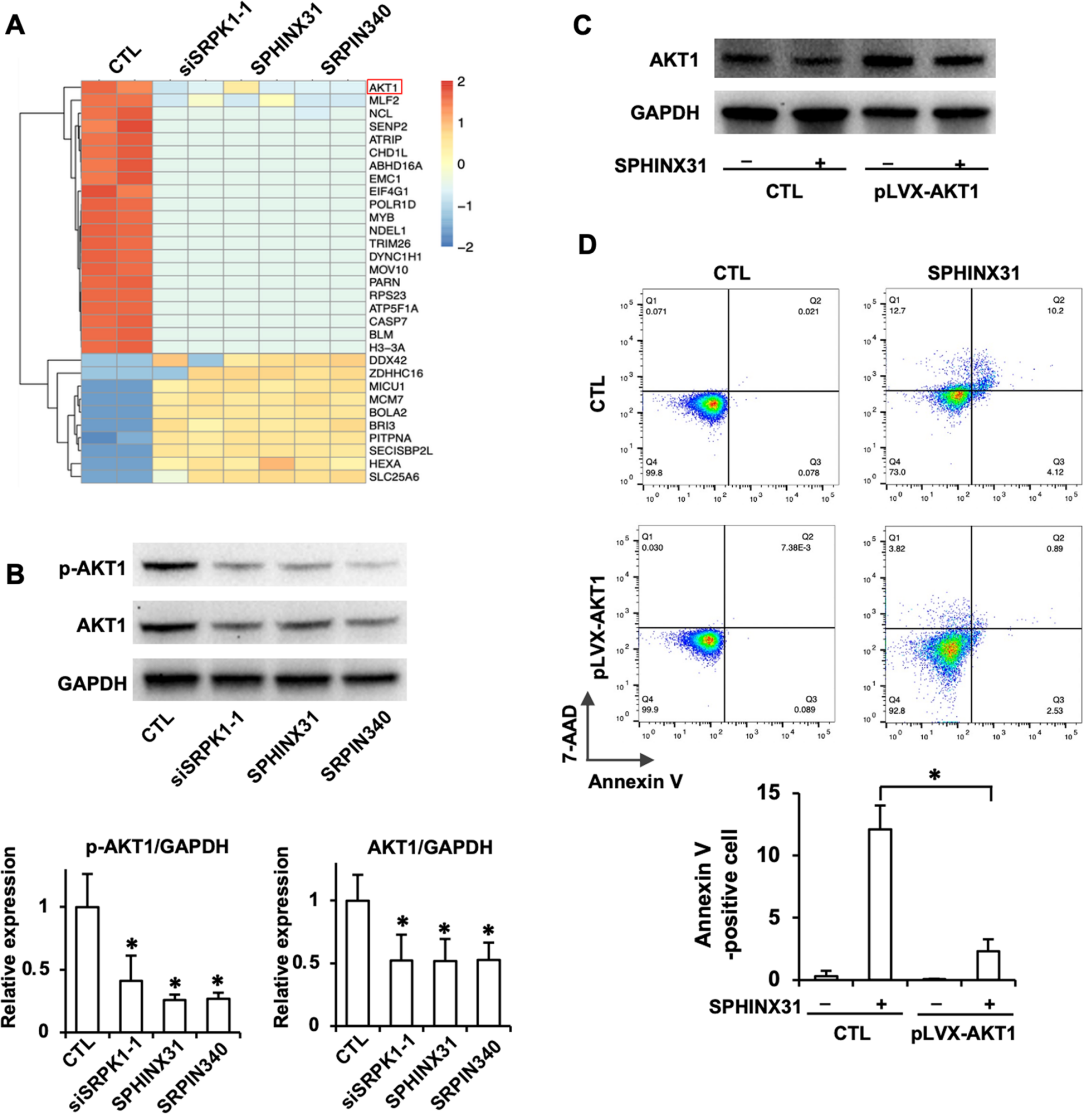
AKT1 is a downstream target of SRPK1 inhibitors in YT cells. **A** YT cells were transfected with SRPK1 siRNA or treated with SPHINX31 (12.5 μM) or SRPIN340 (12.5 μM) for 24 h. Heatmap showed that SRPK1 knockdown and inhibition resulted in similar gene expression patterns, among which AKT1 was downregulated by SRPK1 silencing and inhibition. **B** The levels of total and phosphorylated AKT1 were downregulated by SRPK1 silencing and inhibition. **C** Silencing ATF4 by siRNA transfection decreased the expression of ATF4 in YT cells. AKT1 protein levels in YT cells were upregulated by the overexpression plasmid pLVX-SAKT1. **D** Annexin V/7-AAD double staining showed the effect of AKT1 overexpression on SPHINX31-induced apoptosis in YT cells.

The hierarchical clustering of different transcripts showed that SRPK1 knockdown and inhibition resulted in similar gene expression patterns, and AKT1 (also known as protein kinase B, PKB) was downregulated (Fig. 5A). Decreases in both total and phosphorylated AKT1 levels after SRPK1 inhibition was further confirmed by Western blotting (Fig. 5B). To verify the involvement of AKT1 in SRPK1 inhibition-induced apoptosis, we upregulated the expression of AKT1 with the pLVX-AKT1 expression plasmid (Fig. 5C). Upregulation of AKT1 expression rescued SPHINX31-induced apoptosis in YT cells (Fig. 5D).

These results indicated that SRPK1 inhibition induced cell death by activating the ATF4/CHOP pathway and AKT1 inhibition in ENKTL cells.

### Positive SRPK1 expression in ENKTL patients conferred resistance to cisplatin

Studies on the role of SRPK1 in sensitivity or resistance to cisplatin have yielded conflicting results thus far. The earliest evidence showing that SRPK1 is a cisplatin sensitivity gene came from studies of *S. cerevisiae* and human ovarian carcinoma cells (A2780) [22]. SRPK1 was later confirmed to be a cisplatin sensitivity gene in nonseminomatous germ cell tumors [23]. In contrast , studies using pancreatic, breast, colon, and glioma cell lines [24–26] have shown that the downregulation of SRPK1 results in sensitivity to cisplatin-induced cell death, indicating that SRPK1 is a cisplatin resistance-related gene.

To investigate the effect of SRPK1 on cisplatin sensitivity or resistance in ENKTL, we first evaluated the relationship between SRPK1 expression and sensitivity or resistance to cisplatin treatment. As shown in Table 1, 24 patients with ENKTL were treated with chemotherapy regimens including cisplatin; six patients were SRPK1-negative and 5 of them achieved CR or PR. Another 18 patients were SRPK1-positive, and 12 developed relapsed/refractory disease after receiving cisplatin-containing chemotherapy. Positive expression of SRPK1 was significantly associated with significant cisplatin resistance in ENKTL patients (*P =* 0.033).

### Inhibition of SRPK1 promotes ENKTL cell sensitization to cisplatin

**Fig. 6:**
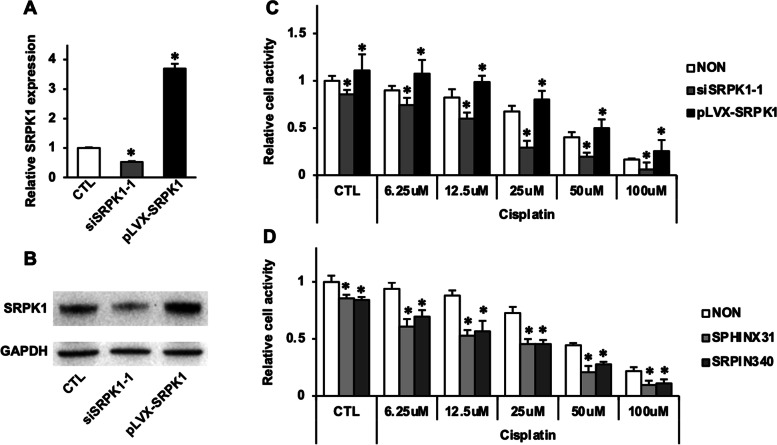
SRPK1 expression conferred resistance to cisplatin in YT cells**.** SRPK1 mRNA (**A**) and protein (**B**) levels in YT cells was down- and upregulated by siSRPK1-1 and the overexpression plasmid pLVX-SRPK1, respectively. **C** The sensitivity of YT cells to cisplatin was decreased and increased upon SRPK1 upregulation and downregulation, respectively (mean activity ± SD from quadruplicate wells, *P < 0.05 versus control group). **D** SPHINX31 and SRPIN340 synergistically enhanced the cytotoxic effects of cisplatin in YT cells (mean activity ± SD from quadruplicate wells, *P < 0.05 compared with the control group).

To further investigate the effects of SRPK1 downregulation and upregulation on cisplatin sensitivity or resistance in ENKTL, SRPK1 was silenced with siRNA or overexpressed with the SRPK1 expression plasmid. We verified that we successfully decreased and increased the mRNA and protein levels of SRPK1 in YT cells (Fig. 6A and B). Compared to normal YT cells, cells with SRPK1 knockdown (siSRPK1-1, gray bars) exhibited significantly increased cisplatin cytotoxicity, while forced SRPK1 overexpression (pLVX-SRPK1, black bars) dramatically decreased the sensitivity of YT cells to cisplatin. In addition, SRPK1 inhibitors (SPHINX31 and SRPIN340) synergistically enhanced the cytotoxic effects of cisplatin on YT cells (Fig. 6C and D). These data demonstrated that SRPK1 is a cisplatin resistance-related protein and that inhibition of SRPK1 promotes ENKTL cells sensitization to cisplatin.

## Discussion

An increasing number studies have shown that aberrant RNA splicing is a widespread phenomenon in cancer and a key event in cancer development [17]. SRPK1, the first and major kinase regulating the alternative splicing of SR splicing factors, can affect several processes in a wide spectrum of malignancies [11–16]. In contrast, studies of SRPK1 in hematopoietic and lymphoid malignancies are rare and mostly focused on leukemias, such as acute adult T-cell leukemia [18], chronic myeloid leukemia [27, 28], acute lymphoblastic leukemia [29], and acute myeloid leukemia [19, 20]. To the best of our knowledge, this is the first study on SRPK1 expression in patients with NK/T-cell lymphoma. In this study, IHC staining of 41 samples and qPCR analyses of 31 samples were performed to evaluate SRPK1 expression in ENKTL. The protein levels of SRPK1 were increased in more than 60% of patients with ENKTL. Additionally, we found that the expression status of SRPK1 was associated with the prognosis; specifically, patients with positive SRPK1 expression experienced significantly shorter OS and PFS than those with negative SRPK1 expression. Positive SRPK1 expression was also shown to confer resistance to cisplatin in ENKTL patients. The notion that SRPK1 is a cisplatin resistance-related protein was corroborated in YT cells, as SRPK1 overexpression by pLVX-SRPK1 plasmid transfection dramatically decreased the sensitivity of YT cells to cisplatin, while SRPK1 downregulation significantly increased cisplatin cytotoxicity. We further demonstrated that both SRPK1 inhibitors (SPHINX31 and SRPIN340) suppressed cell proliferation and promoted apoptosis in YT cells and PBLs from ENKTL patients. Furthermore, RNA-seq enrichment analysis and Western blotting showed that the ATF4/CHOP/AKT1 axis was a downstream target of SRPK1 inhibitors in YT cells.

The relationship between SRPK1 expression and sensitivity or resistance to cisplatin-containing chemotherapy appeared to be tumor/cell specific. For example, although positive expression of SRPK1 was associated with resistance to cisplatin-containing chemotherapy in ENKTL patients, similar to the pancreatic carcinoma cell lines reported by Hayes GM *et a*l. [24], the opposite effect was observed by Schenk *et al.* in ovarian carcinoma cell lines [22] and germ cell tumors [23]. The hypothesis that SRPK1 was experimentally proven to be a cisplatin resistance-related protein in the YT cell line based on the results of a cisplatin killing experiment (Fig. 6); thus, we postulate that the discrepant results between these studies may be due to intrinsic differences between different types of tissue/cells and/or differences in the expression of targeted genes regulated by SRPK1 through RNA splicing in different types of cancer. It is of paramount importance to assess the expression status of each tumor to accordingly select the most effective chemotherapy regimen. Although large-scale prospective studies with more patients are needed, based on our limited and small cohort of ENKTL patients, an alternative chemotherapy regimen without cisplatin should be selected for the treatment of ENKTL when positive SRPK1 expression is detected. Interestingly, the relationship between the expression status of SRPK1 and sensitivity to cisplatin is dynamic and not static, at least in cell lines, as revealed by Wang C *et al.,* as cisplatin-resistant cells were resensitized by inhibiting the kinase activity or enhancing the acetylation of SRPK1 [30]. In addition, aberrantly expressed SRPK1 can regulate diverse oncogenic processes, including angiogenesis, cell proliferation, and apoptosis, through various mechanisms/pathways, and SRPK1 inhibition results in changes in the transcript levels of many genes, highlighting the potential possibility that SRPK1 itself can serve as a therapeutic target [11, 31].

While the mechanism(s) through which SRPK1 can serve as a prospective treatment target remain unclear, the RNA-seq analysis in this study suggests at least a possible partial explanation. As shown in the heatmap and GO enrichment analysis data, inhibition of SRPK1 in YT cells led to increased ATF4 and CHOP expression and decreased AKT1 expression. The ATF4-CHOP signaling pathway may play an essential role in the increased apoptosis induced by SRPK1 inhibition. Studies have revealed the importance of the ATF4/CHOP pathway in the LW-213-induced apoptosis of cutaneous T-cell lymphoma cells [32] and the aspirin-induced apoptosis in multiple myeloma cells [33]. ERS-mediated apoptosis was observed in gambogic acid-treated ENKTL cells, which was triggered by activation of the ATF4/CHOP pathway and inhibition of the AKT [34]. Similar to our results, ATF4/CHOP activation and AKT inhibition were observed in SRPK1 inhibitor-treated YT cells. Huang D *et al.* reported that the oncogenic phosphatidylinositol 3-kinase (PIK3)/AKT pathway was abnormally activated in patients with PTCL and ENKTL, and a PI3K inhibitor induced cell cycle arrest but not the apoptosis in PTCL and ENKTL cells [35]. In this study, the suppression of AKT1 induced by SRPK1 inhibition may have induced apoptosis or cell cycle arrest, resulting in the suppression of ENKTL cell proliferation. The AKT1 pathway was reported to be associated with chemoresistance in cancers [36, 37], overexpression of phosphor-AKT1 and AKT1 was observed in cisplatin-resistant cancer cells [38, 39], and downregulation of AKT was shown to reverse cisplatin resistance in osteosarcoma cells [40] and non-small‐cell lung cancer cells [41, 42]. AKT1 suppression induced by SRPK1 inhibition may play an important role in restoring cisplatin sensitivity in ENKTL cells. Further experiments are required to explore the precise mechanism of SRPK1-mediated cisplatin resistance in ENKTL.

In summary, we report that SRPK1 overexpression is associated with an adverse prognosis and cisplatin resistance in ENKTL patients. SRPK1 inhibition or silencing suppresses cell proliferation and promotes apoptosis, most likely at least in part by activating the ATF4-CHOP pathway and suppressing AKT1 expression. Our studies may pave the way for future studies on SRPK1 as a potential therapeutic target for ENKTL.

## Supplementary Information

**Additional file 1.**

**Additional file 2.**

## Data Availability

The RNA-seq datasets generated during the current study are available in the NCBI SRA database (https://www.ncbi.nlm.nih.gov/bioproject/PRJNA774953).

## Abbreviations

ENKTL: Extranodal natural killer/T-cell lymphoma
SRPK1: Serine/arginine-rich protein kinase 1
PTCL: Peripheral T-cell lymphoma
AML: Acute myeloid leukemia
FFPE: Formalin-fixed paraffin-embedded
IHC: Immunohistochemistry
PBLs: Peripheral blood lymphocytes
GAPDH: Glyceraldehyde-3-phosphate dehydrogenase
siRNA: Small interfering RNA
7-AAD: 7-Amino-actinomycin D
PARP: Poly-ADP-ribose polymerase
ATF-4: Activating transcription factor 4
CHOP: C/EBP homologous protein
AKT1: Protein kinase B, PKB
OS: Overall survival
PFS: Progression-free survival
LDH: Lactate dehydrogenase
GO: Gene Ontology
PI3K: Phosphatidylinositol 3-kinase

## References

[1] Fox CP, Civallero M, Ko YH, Manni M, Skrypets T, Pileri S, Kim SJ, Cabrera ME, Shustov AR, Chiattone CS (2020). Survival outcomes of patients with extranodal natural-killer T-cell lymphoma: a prospective cohort study from the international T-cell Project. Lancet Haematol..

[2] Tse E, Kwong YL (2017). The diagnosis and management of NK/T-cell lymphomas. J Hematol Oncol..

[3] Inoue D, Chew GL, Liu B, Michel BC, Pangallo J, D'Avino AR, Hitchman T, North K, Lee SC, Bitner L (2019). Spliceosomal disruption of the non-canonical BAF complex in cancer. Nature..

[4] Seiler M, Peng S, Agrawal AA, Palacino J, Teng T, Zhu P, Smith PG, Buonamici S, Yu L (2018). Somatic Mutational Landscape of Splicing Factor Genes and Their Functional Consequences across 33 Cancer Types. Cell Rep..

[5] Shuai S, Suzuki H, Diaz-Navarro A, Nadeu F, Kumar SA, Gutierrez-Fernandez A, Delgado J, Pinyol M, Lopez-Otin C, Puente XS (2019). The U1 spliceosomal RNA is recurrently mutated in multiple cancers. Nature..

[6] Fu XD (1995). The superfamily of arginine/serine-rich splicing factors. RNA..

[7] Manley JL, Krainer AR (2010). A rational nomenclature for serine/arginine-rich protein splicing factors (SR proteins). Genes Dev..

[8] Gui JF, Lane WS, Fu XD (1994). A serine kinase regulates intracellular localization of splicing factors in the cell cycle. Nature..

[9] Zhou Z, Fu XD (2013). Regulation of splicing by SR proteins and SR protein-specific kinases. Chromosoma..

[10] Ding JH, Zhong XY, Hagopian JC, Cruz MM, Ghosh G, Feramisco J, Adams JA, Fu XD (2006). Regulated cellular partitioning of SR protein-specific kinases in mammalian cells. Mol Biol Cell..

[11] Nikas IP, Themistocleous SC, Paschou SA, Tsamis KI, Ryu HS. Serine-Arginine Protein Kinase 1 (SRPK1) as a Prognostic Factor and Potential Therapeutic Target in Cancer: Current Evidence and Future Perspectives. Cells. 2019;9 (1): 10.3390/cells9010019 10.3390/cells9010019 PMC7017105 31861708

[12] Dong Z, Chang X, Xie L, Wang Y, Hou Y (2022). Increased expression of SRPK1 (serine/arginine-rich protein-specific kinase 1) is associated with progression and unfavorable prognosis in cervical squamous cell carcinoma. Bioengineered..

[13] van Roosmalen W, Le Devedec SE, Golani O, Smid M, Pulyakhina I, Timmermans AM, Look MP, Zi D, Pont C, de Graauw M (2015). Tumor cell migration screen identifies SRPK1 as breast cancer metastasis determinant. J Clin Invest..

[14] Yao Y, Li Q, Wang H. MiR-216b suppresses colorectal cancer proliferation, migration, and invasion by targeting SRPK1. Onco Targets Ther. 2018;11(1671–1681. 10.2147/OTT.S161835 10.2147/OTT.S161835 PMC5870636 29615842

[15] Li Y, Yu S, Wang X, Ye X, He B, Quan M, Gao Y (2019). SRPK1 facilitates tumor cell growth via modulating the small nucleolar RNA expression in gastric cancer. J Cell Physiol..

[16] Wang F, Zhou J, Xie X, Hu J, Chen L, Hu Q, Guo H, Yu C (2015). Involvement of SRPK1 in cisplatin resistance related to long non-coding RNA UCA1 in human ovarian cancer cells. Neoplasma.

[17] Obeng EA, Stewart C, Abdel-Wahab O (2019). Altered RNA Processing in Cancer Pathogenesis and Therapy. Cancer Discov..

[18] Hishizawa M, Imada K, Sakai T, Ueda M, Hori T, Uchiyama T (2005). Serological identification of adult T-cell leukaemia-associated antigens. Br J Haematol..

[19] Tzelepis K, De Braekeleer E, Aspris D, Barbieri I, Vijayabaskar MS, Liu WH, Gozdecka M, Metzakopian E, Toop HD, Dudek M (2018). SRPK1 maintains acute myeloid leukemia through effects on isoform usage of epigenetic regulators including BRD4. Nat Commun..

[20] Tzelepis K, Koike-Yusa H, De Braekeleer E, Li Y, Metzakopian E, Dovey OM, Mupo A, Grinkevich V, Li M, Mazan M (2016). A CRISPR Dropout Screen Identifies Genetic Vulnerabilities and Therapeutic Targets in Acute Myeloid Leukemia. Cell Rep..

[21] Swerdlow SH, Campo E, Pileri SA, Harris NL, Stein H, Siebert R, Advani R, Ghielmini M, Salles GA, Zelenetz AD (2016). The 2016 revision of the World Health Organization classification of lymphoid neoplasms. Blood..

[22] Schenk PW, Boersma AW, Brandsma JA, den Dulk H, Burger H, Stoter G, Brouwer J, Nooter K (2001). SKY1 is involved in cisplatin-induced cell kill in Saccharomyces cerevisiae, and inactivation of its human homologue, SRPK1, induces cisplatin resistance in a human ovarian carcinoma cell line. Cancer Res..

[23] Schenk PW, Stoop H, Bokemeyer C, Mayer F, Stoter G, Oosterhuis JW, Wiemer E, Looijenga LH, Nooter K (2004). Resistance to platinum-containing chemotherapy in testicular germ cell tumors is associated with downregulation of the protein kinase SRPK1. Neoplasia..

[24] Hayes GM, Carrigan PE, Beck AM, Miller LJ (2006). Targeting the RNA splicing machinery as a novel treatment strategy for pancreatic carcinoma. Cancer Res..

[25] Hayes GM, Carrigan PE, Miller LJ (2007). Serine-arginine protein kinase 1 overexpression is associated with tumorigenic imbalance in mitogen-activated protein kinase pathways in breast, colonic, and pancreatic carcinomas. Cancer Res..

[26] Sigala I, Tsamis KI, Gousia A, Alexiou G, Voulgaris S, Giannakouros T, Kyritsis AP, Nikolakaki E (2016). Expression of SRPK1 in gliomas and its role in glioma cell lines viability. Tumour Biol..

[27] Salesse S, Dylla SJ, Verfaillie CM (2004). p210BCR/ABL-induced alteration of pre-mRNA splicing in primary human CD34+ hematopoietic progenitor cells. Leukemia..

[28] Wang H, Ge W, Jiang W, Li D, Ju X (2018). SRPK1siRNA suppresses K562 cell growth and induces apoptosis via the PARPcaspase3 pathway. Mol Med Rep..

[29] Siqueira RP, Caetano MMM, de Souza LA, Dos Passos PMS, Simaroli NB, Barros MVA, de Souza APM, de Oliveira LL, Silva-Junior A, Fietto JLR *et al*. Combined SRPK and AKT pharmacological inhibition is synergistic in T-cell acute lymphoblastic leukemia cells. Toxicol In Vitro. 2020;65 (104777. 10.1016/j.tiv.2020.104777 10.1016/j.tiv.2020.104777 31962201

[30] Wang C, Zhou Z, Subhramanyam CS, Cao Q, Heng ZSL, Liu W, Fu X, Hu Q (2020). SRPK1 acetylation modulates alternative splicing to regulate cisplatin resistance in breast cancer cells. Commun Biol..

[31] Bullock N, Oltean S (2017). The many faces of SRPK1. J Pathol..

[32] Yu XX, Zhu MY, Wang JR, Li H, Hu P, Qing YJ, Wang XY, Wang HZ, Wang ZY, Xu JY (2021). LW-213 induces cell apoptosis in human cutaneous T-cell lymphomas by activating PERK-eIF2alpha-ATF4-CHOP axis. Acta Pharmacol Sin..

[33] Liu H, Xiong C, Liu J, Sun T, Ren Z, Li Y, Geng J, Li X. Aspirin exerts anti-tumor effect through inhibiting Blimp1 and activating ATF4/CHOP pathway in multiple myeloma. Biomed Pharmacother. 2020;125(110005. 10.1016/j.biopha.2020.110005 10.1016/j.biopha.2020.110005 32070879

[34] Peng W, Chen BA (2018). Gambogic acid induces cell apoptosis through endoplasmic reticulum stress triggered inhibition of Akt signaling pathways in extranodal NK/T-cell lymphoma cells. Chin J Nat Med..

[35] Huang D, Song TL, Nairismagi ML, Laurensia Y, Pang WL, Zhe DCM, Wong EKY, Wijaya GG, Tan J, Tan SH (2020). Evaluation of the PIK3 pathway in peripheral T-cell lymphoma and NK/T-cell lymphoma. Br J Haematol..

[36] Kim D, Dan HC, Park S, Yang L, Liu Q, Kaneko S, Ning J, He L, Yang H, Sun M (2005). AKT/PKB signaling mechanisms in cancer and chemoresistance. Front Biosci..

[37] Pommier Y, Sordet O, Antony S, Hayward RL, Kohn KW (2004). Apoptosis defects and chemotherapy resistance: molecular interaction maps and networks. Oncogene..

[38] Sen T, Sen N, Brait M, Begum S, Chatterjee A, Hoque MO, Ratovitski E, Sidransky D (2011). DeltaNp63alpha confers tumor cell resistance to cisplatin through the AKT1 transcriptional regulation. Cancer Res..

[39] Tang C, Luo H, Luo D, Yang H, Zhou X (2018). Src homology phosphotyrosyl phosphatase 2 mediates cisplatin-related drug resistance by inhibiting apoptosis and activating the Ras/PI3K/Akt1/survivin pathway in lung cancer cells. Oncol Rep..

[40] Zhao L, Zhang W, Zhang F (2021). Poncirin downregulates ATP-binding cassette transporters to enhance cisplatin sensitivity in cisplatin-resistant osteosarcoma cells. Phytother Res..

[41] Dukaew N, Chairatvit K, Pitchakarn P, Imsumran A, Karinchai J, Tuntiwechapikul W, Wongnoppavich A (2020). Inactivation of AKT/NFkappaB signaling by eurycomalactone decreases human NSCLC cell viability and improves the chemosensitivity to cisplatin. Oncol Rep..

[42] Zhang S, Wang Y (2020). Deoxyshikonin inhibits cisplatin resistance of non-small-cell lung cancer cells by repressing Akt-mediated ABCB1 expression and function. J Biochem Mol Toxicol..

