# Supplementary material for: Inhibition of SRPK1, a key splicing regulator, exhibits antitumor and chemotherapeutic-sensitizing effects on extranodal NK/T-cell lymphoma cells
Source: BMC Cancer. 2022 Oct 27;22:1100. doi: 10.1186/s12885-022-10158-6 (PMC9609466; doi:10.1186/s12885-022-10158-6)
Supplement: Supplementary file 1 — Additional file 1. [file 12885_2022_10158_MOESM1_ESM.pdf]

Sup Figure. 1

a SRPK1 Negative Control

b SRPK1 Positive Control

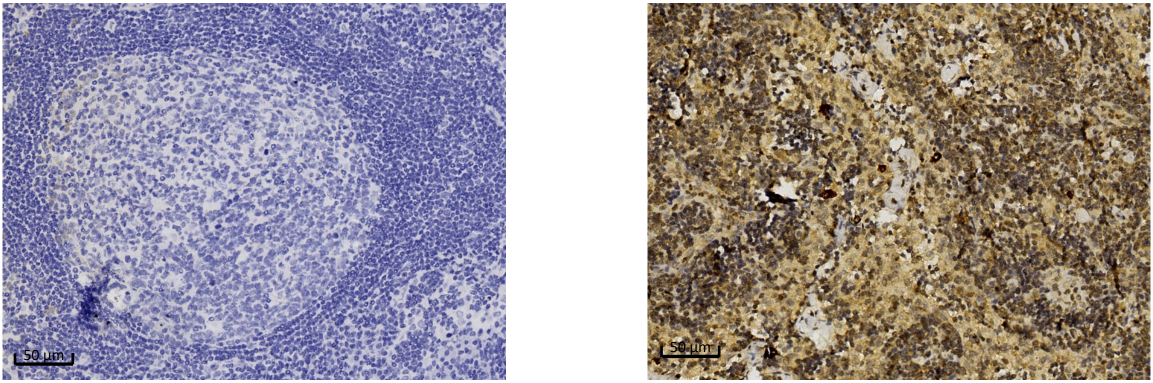

Normal lymph node and ovarian carcinoma tissues were used as negative and positive controls, respectively.
