# Supplementary figures and images for: Inhibition of SRPK1, a key splicing regulator, exhibits antitumor and chemotherapeutic-sensitizing effects on extranodal NK/T-cell lymphoma cells
Source: BMC Cancer. 2022 Oct 27;22:1100. doi: 10.1186/s12885-022-10158-6 (PMC9609466; doi:10.1186/s12885-022-10158-6)

Figure. 2B

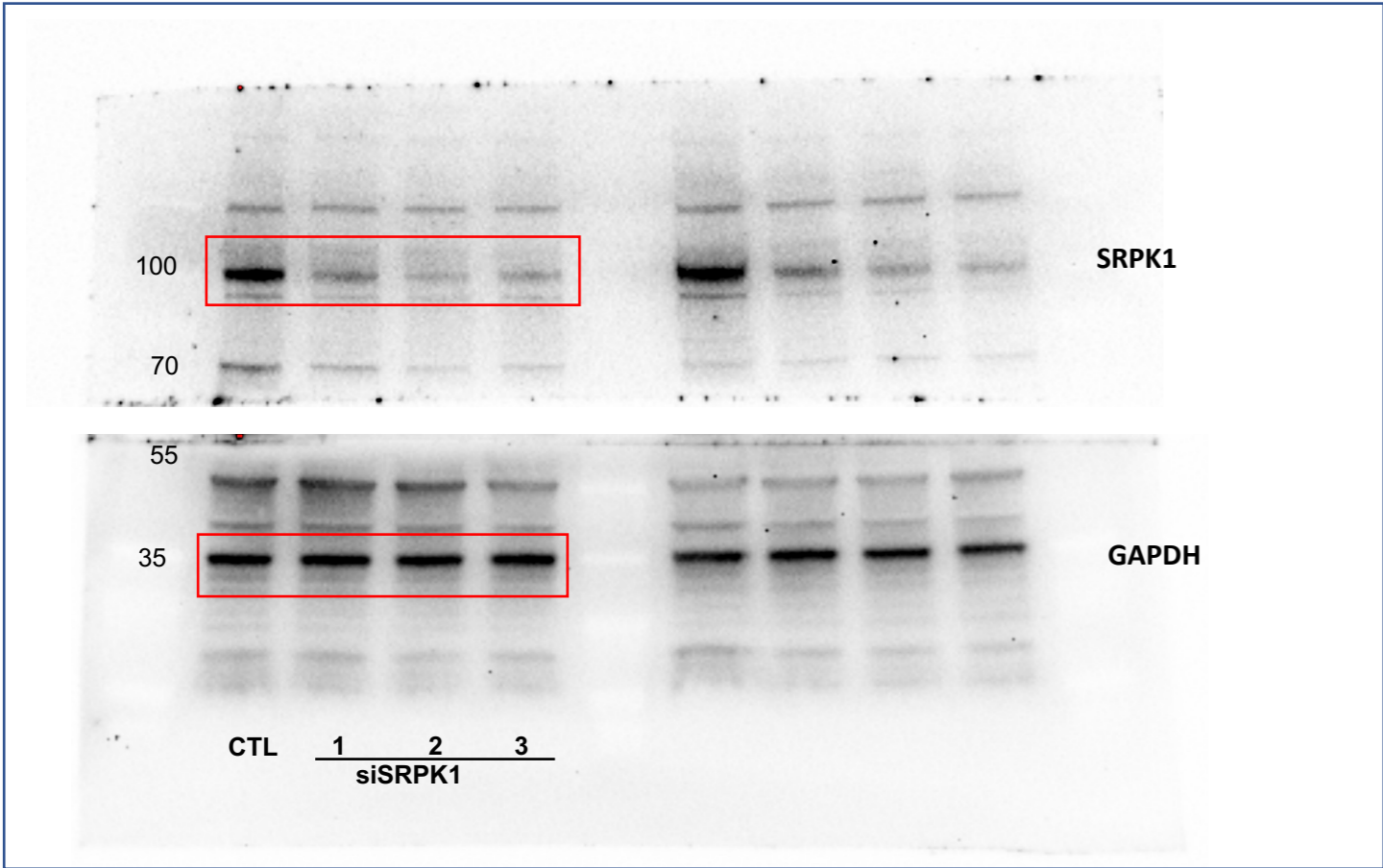

Figure. 3B

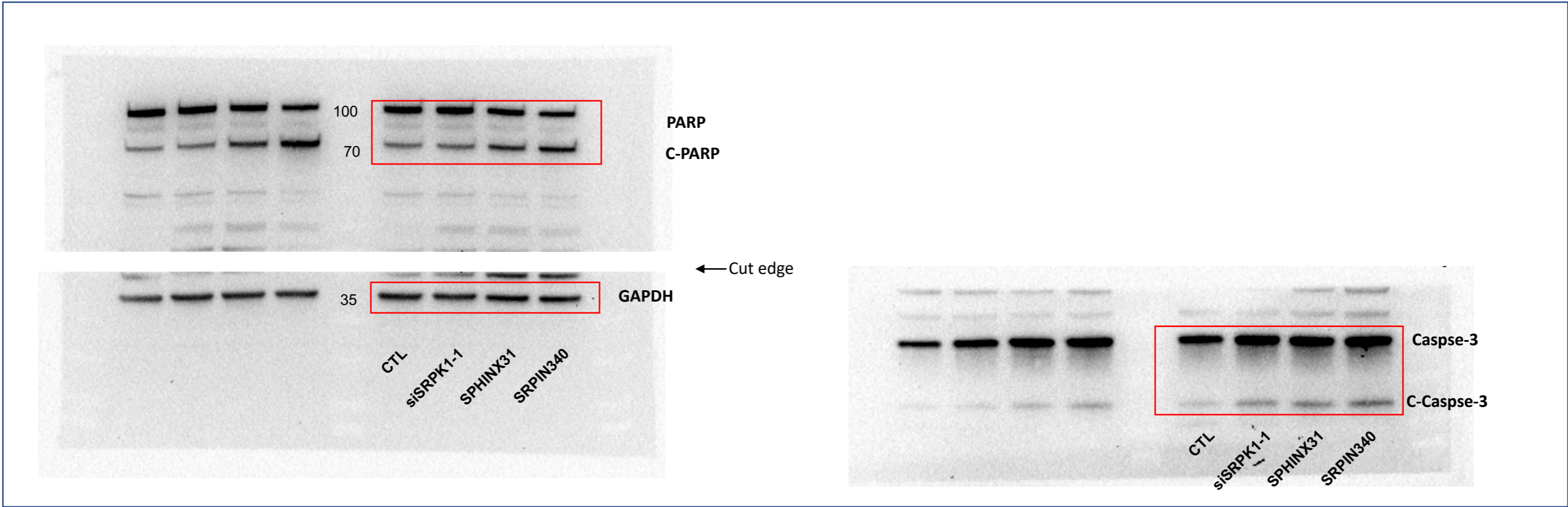

Figure. 4B

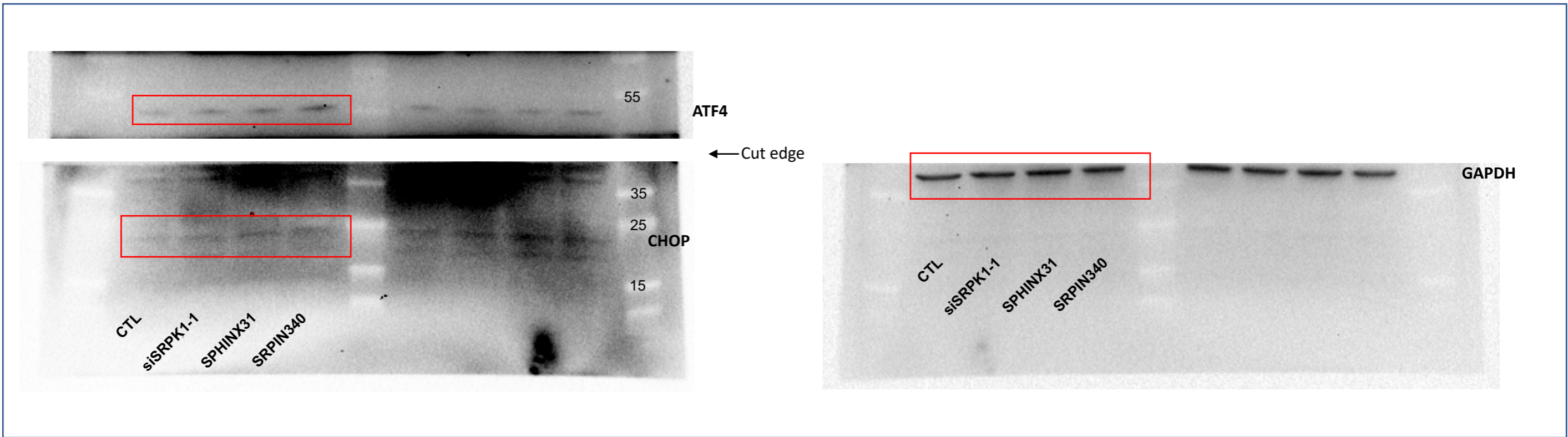

Figure. 4C

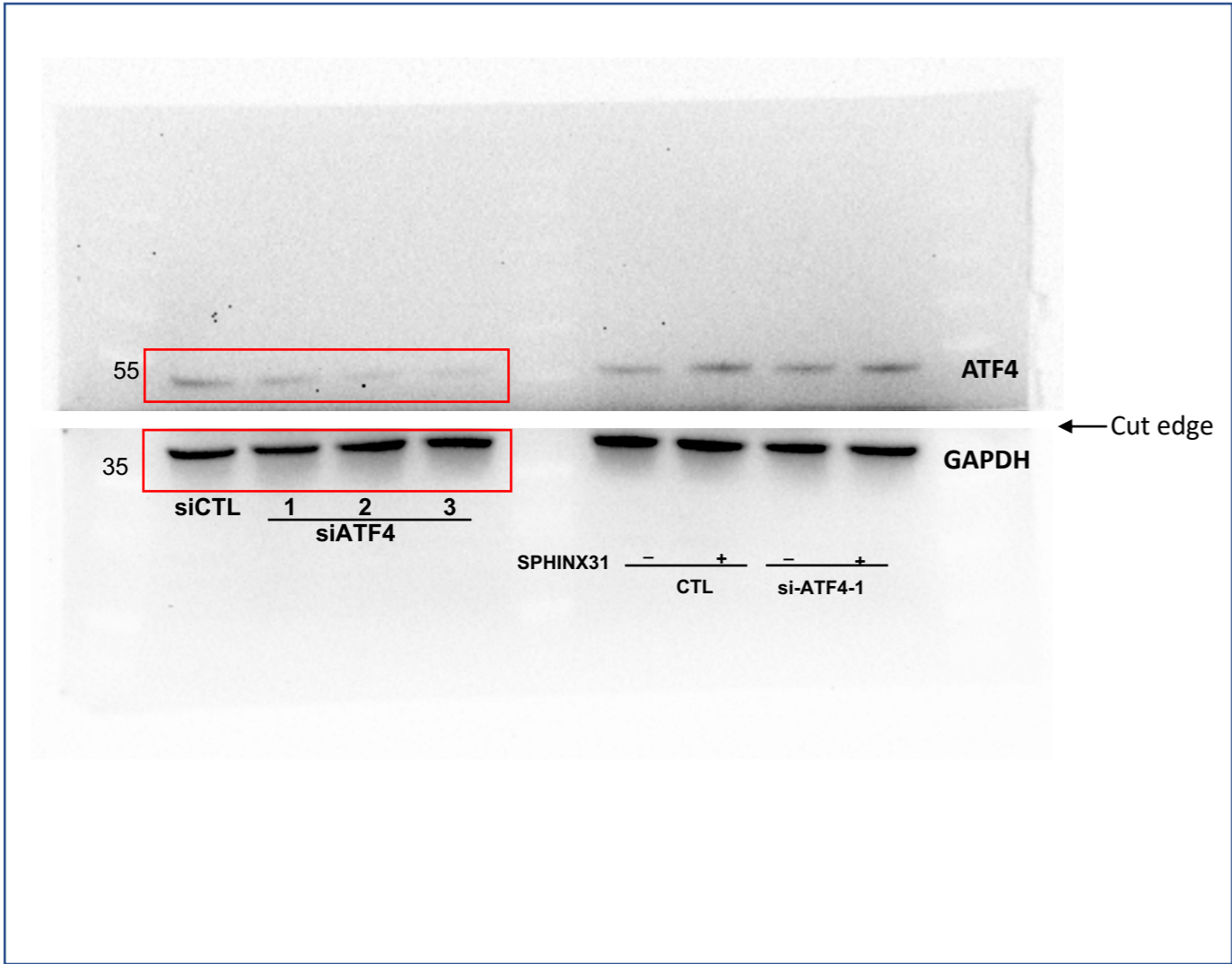

Figure. 5B

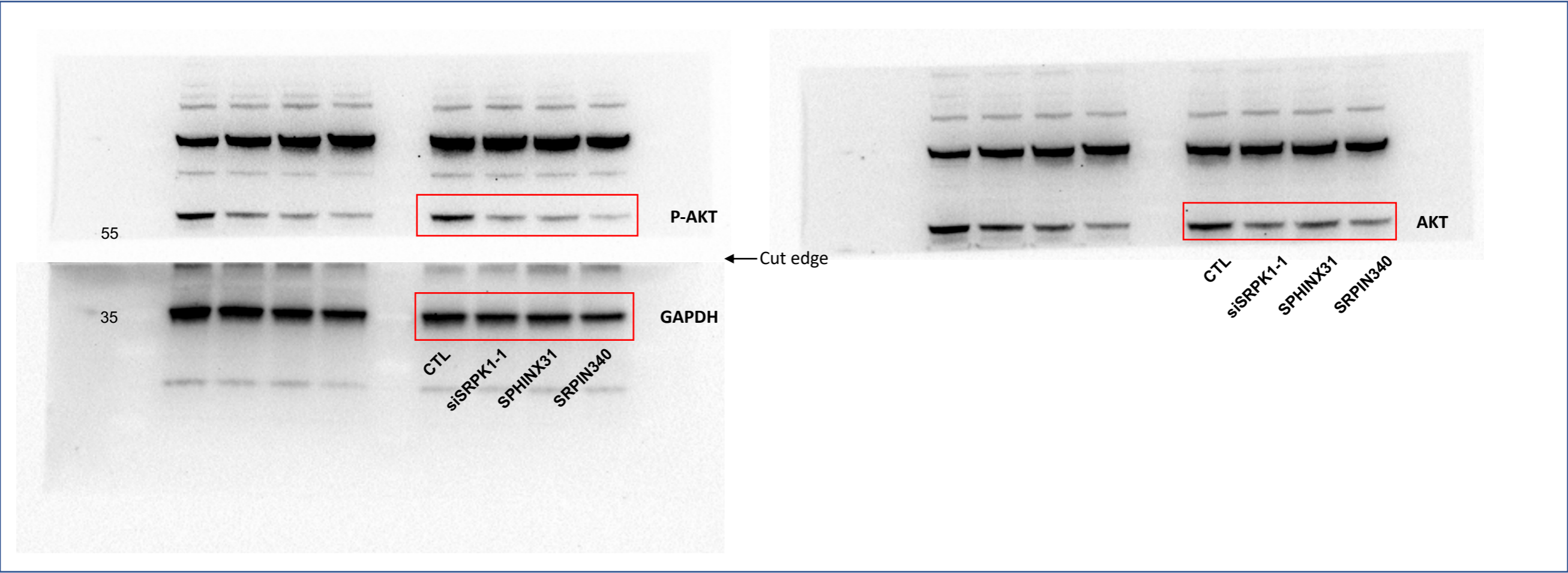

Figure. 5C

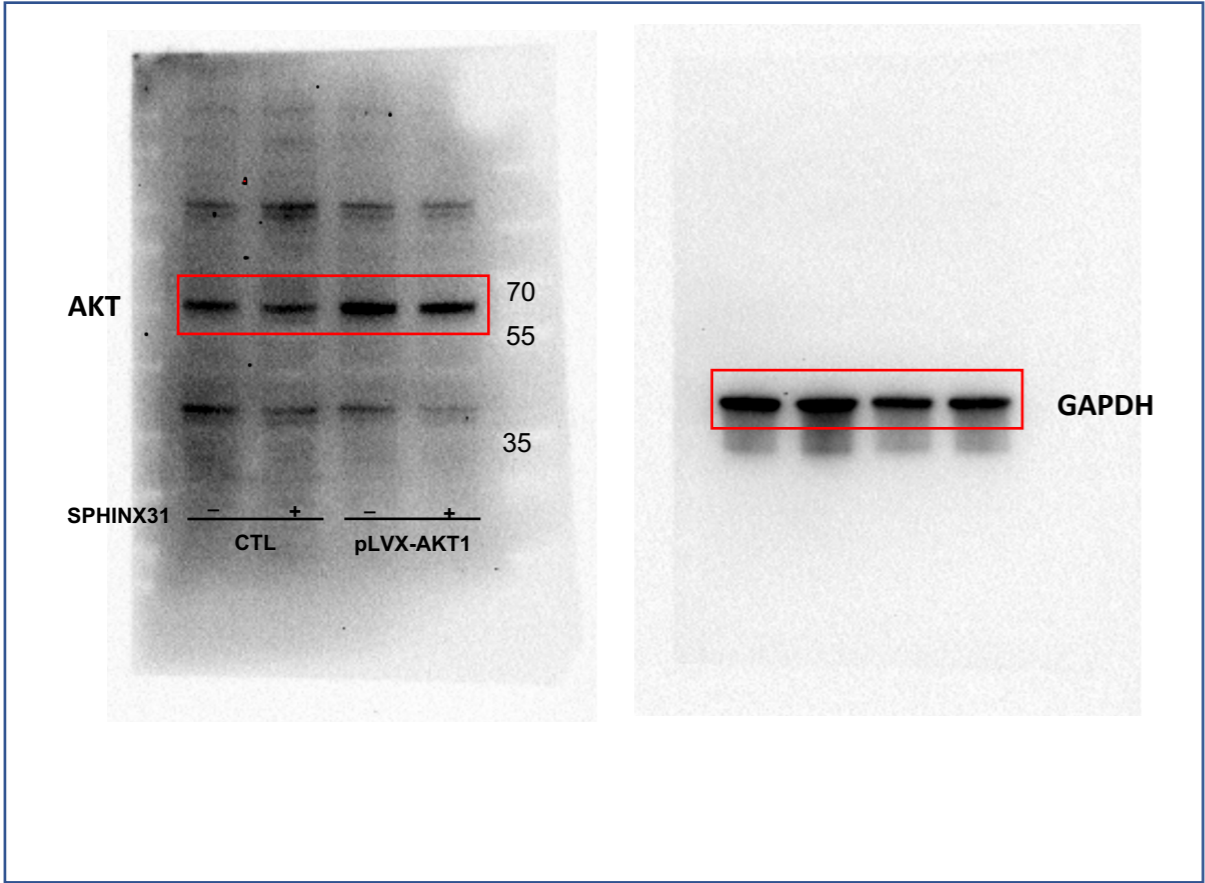

Figure. 6B

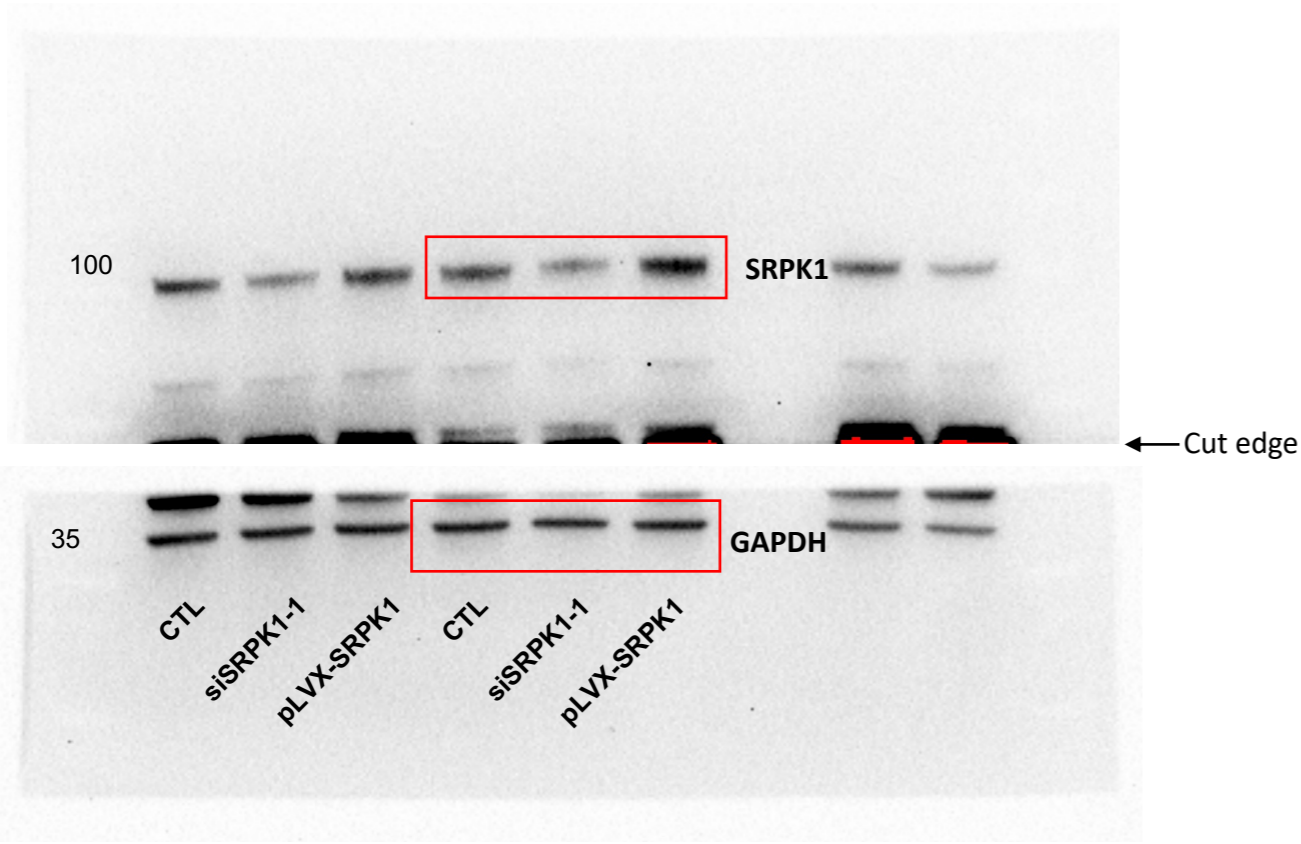

Supplement: Supplementary file 2 — Additional file 2. [file 12885_2022_10158_MOESM2_ESM.pdf]
